# Editorial: The Role of Environmental Reservoirs in *Campylobacter*-Mediated Infection

**DOI:** 10.3389/fcimb.2021.773436

**Published:** 2021-10-21

**Authors:** Shymaa Enany, Alessandra Piccirillo, Mohamed Elhadidy, Piotr Tryjanowski

**Affiliations:** ^1^ Microbiology and Immunology Department, Faculty of Pharmacy, Suez Canal University, Ismailia, Egypt; ^2^ Biomedical Research Department, Armed Force College of Medicine, Cairo, Egypt; ^3^Department of Comparative Biomedicine and Food Science, University of Padua, Padova, Italy; ^4^Biomedical Sciences Program, University of Science and Technology, Zewail City of Science and Technology, Giza, Egypt; ^5^Department of Bacteriology, Mycology and Immunology, Faculty of Veterinary Medicine, Mansoura University, Mansoura, Egypt; ^6^Faculty of Veterinary Medicine & Animal Sciences, Poznan University of Life Sciences, Poznan, Poland; ^7^Faculty of Environmental Sciences, Czech University of Life Sciences, Prague, Czech Republic

**Keywords:** campylobacteriosis, biofilm formation, *Campylobacter* ecology, whole genome sequencing, infection

Campylobacteriosis is a food-borne infection caused mainly by *Campylobacter jejuni* (*C. jejuni*) and *Campylobacter coli (C. coli)*. Thermophilic campylobacters are considered the foremost causative agents of bacterial gastroenteritis worldwide and campylobacteriosis represents an important public health problem with numerous socio-economic impacts. Since 2015, approximately 230,000 cases have been reported annually in Europe (Hansson et al., 2018). *C. jejuni* and *C. coli* colonize the digestive tract of multiple animal reservoirs such as birds, sheep, cattle, and pigs, with chicken considered as the main source of human infection in many countries. Yet, epidemiological information regarding the role of other putative disease reservoirs, such as wildlife and environment (*e.g.* soil and water), in disease transmission is limited.

Although *Campylobacter* can grow only inside the animal host, these bacteria can develop survival strategies by adaptation to different environmental stresses such as fluctuations of oxygen, temperature, biotic interactions, and starvation (Mouftah et al., 2021b). Moreover, the biofilm formation and the interaction with other bacteria also affects the bacterial survival outside the host. Studying the biology of* Campylobacter* in different environments may provide a better understanding of the infectivity of surviving bacteria to human.

The review by Kim et al. focused on molecular mechanisms involved in *Campylobacter* resistance to adverse environmental conditions, such as acid (*e.g.* ATR initiation, chaperone proteins DnaK and GroEL, and *rpoS* gene) and thermal stress (*e.g.* cold shock protein CspA), high osmolarity (*e.g. RpoN* sigma factor), as well as to antimicrobial agents (*e.g.* tetracyclines, quinolones, and macrolides). In the food production chain, *Campylobacter* is thought to be particularly susceptible to oxidative and desiccation stresses; however, *C. jejuni* has been demonstrated to be able to adapt to aerobic atmosphere by activating a range of enzymes, including KatA catalase. Thermophilic campylobacters are also able to respond to low temperatures encountered in food processing, even though the growth is particularly slow down. One of the food sources allowing a long-term survival of *C. jejuni* is the “chicken juice”, in which it is able to form biofilm after expression of the quorum sensing system activated by the *luxS* gene. Under stressful conditions, *C. jejuni* is also able to enter a viable but non-culturable (VBNC) state that, among the others, affects CadF expression and thus its invasion ability.

Molecular strategies used by *Campylobacter* to survive in environmental and/or under stress conditions were also reviewed by Elmi et al. who highlighted the need to employ integrative multi-omics and phenotypic assays in research studies aimed to understand *C. jejuni* pathogenesis in multiple hosts. When exposed to high oxygen tension, limited nutrient availability, heat, acidic pH, temperatures fluctuations and antimicrobials, *Campylobacter* response is mainly due to its (strain-dependent) ability to form biofilms and to switch to the VBNC state. *Campylobacter* does not possess the same genetic repertoire of other bacteria to sense, adapt and survive to stress. *Campylobacter’s* ability to form biofilm is thought to be under the control of a complex array of regulatory factors (*e.g.* CsrA, CosR, SpotT and CprRS), whereas heat shock proteins (HSPs) seem to be involved in *C. jejuni* response to temperature stress, and two-component regulatory systems (TCSs), RacRS, and RpoN, to acid stress. Recent studies employing whole genome sequencing (WGS) have suggested that *C. jejuni* host adaptation might be related to *panBCD* genes. Additionally, the *C. jejuni* T6SS seems to play a fundamental role in pathogenesis. *Campylobacter* flagella are well known factors involved in multiple functions including virulence, and recent studies have demonstrated that even minor genetic modifications may influence host colonization and infection. In addition, other factors involved in *Campylobacter* pathogenicity, such as capsule polysaccharide (CPS), adhesins (*e.g.* CadF and FlpA), and major outer membrane proteins (MOMPs) still need to be further explored. *C. jejuni* pathogenicity factors, such as outer membrane vesicles (OMVs), have been recently suggested as important fitness and survival factors as well. Finally, *Campylobacter* has the unique ability to metabolize only a few amino acids that seems to be the main strategy supporting its survival and adaptation in hosts.

Poultry and their products are the main reservoir of *Campylobacter* spp. relevant for public health, and Hakeem and Lu reviewed their transmission, survival, and adaptation in poultry production environments, including farms and processing systems. *Campylobacter* is reported to be widespread in these settings and many sources (soil, water, air, insects, animals, humans, several processing steps, *e.g.* scalding, defeathering, evisceration, neck removal, inside and outside washing) may contribute to its introduction and dissemination along the poultry production chain. Thus, improving on-farm biosecurity and in-plant control strategies are key elements to limit the prevalence of *Campylobacter* in poultry farms and products. One of the most striking aspects of the relationship between poultry and *Campylobacter* is gut colonization occurring at very high levels (100%) and nothing seems to be effective in reducing it, suggesting a commensal relationship between them. Different intervention strategies (*e.g.* vaccination, phage therapy, bacteriocins, probiotics, fatty acids, and essential oils) have been investigated to control *Campylobacter* colonization without any conclusive findings. Currently, only interventions in poultry processing plants seem to decrease the chance of *Campylobacter* contamination of poultry meat and consequently human infection. In their review, Hakeem and Lu presented novel and alternative strategies (*e.g.* plant-based antimicrobials, metal oxide nanoparticles, and antimicrobial synergism) to prevent and control *Campylobacter* in the agro-ecosystem.

However, domesticated birds are not the only source of *Campylobacter*, also wild birds are important source of human enteric pathogens, including bacteria of the genus *Campylobacter*, occurring in their digestive tracts. Interestingly, these species may be vectors of antimicrobial resistance (AMR) in the environment due to contact with antibiotics (Tryjanowski et al., 2020). Therefore, new studies focused on understanding *Campylobacter* sources, visible in this sample of published papers, including development of new molecular diagnostic methods (Carraro et al., 2019; Mouftah et al., 2021a; Saif et al., 2021) and will be useful with prevention (Facciolà et al., 2017).

Morcrette et al. focused on persister cell formation as mechanism used by *C. jejuni* to survive to stress induced by bactericidal concentrations of antibiotics. *C. jejuni* showed the ability to form persister cells at a frequency of 10^−3^ after exposure to 100 × MIC of penicillin G for 24 h. Metabolic activity detected by Redox Sensor Green reagent (RSG) staining suggested that this ability may be the consequence of increased redox protein activity in, or associated with, the electron transport chain. Proteomic analysis showed increased levels of redox proteins, such as reductases, in cells exposed to the antibiotic and indicated a remodelling of the electron transfer chain toward a less electrogenic process in order to moderate membrane hyperpolarization and intracellular alkalization; thus, reducing the antibiotic efficacy and potentially assisting in persister cell formation.

Shagieva et al. investigated the different ability of adhesion and biofilm formation of *C. jejuni* isolates from different sources (surface and wastewater, food, and clinical samples), as well as the potential role of the *luxS* gene (responsible for production of the communication molecule AI-2) in biofilm formation. All *C. jejuni* isolates were able to adhere to a surface, whereas the quantity and the architecture of biofilms were diverse, with wastewater isolates forming more compact biofilms. Not all isolates possessed the *luxS* gene, in particular those originated from surface waters. These isolates formed thinner and sparser biofilms lacking the presence of significant clusters. However, the ability to adhere to the surface was preserved. Overall, this study showed that *C. jejuni* isolated from water can adhere to a surface and subsequently form a spatially structured biofilm. As their adhesion capacity was comparable to the strains of clinical or food origin, they might indeed represent a significant source of contamination in animal husbandry, and as a source of infection in humans.

Although comparative genomic analysis using conventional seven-locus multilocus sequence typing (MLST) has been used as the gold standard over the past decades in attributing different reservoirs of this foodborne pathogen, WGS-based methods enable a better understanding of *Campylobacter* epidemiology, multi-host ecology, host adaptation, and cryptic transmission networks from animals to humans at the farm-to-fork interface through providing higher-resolution typing, that was not possible to achieve with other previous typing methods. Furthermore, microbial genomics has been recently applied to forecast the genetic determinants involved in *Campylobacter* antimicrobial resistance to detect the complex dynamics of selection and transmission of AMR through a multidisciplinary One Health approach (Mouftah et al., 2021a).

For instance, Nennig at al. implemented WGS gene-by-gene approach to better understand *Campylobacter* epidemiology. In this pilot study, they highlighted the clonal expansion of stable genomes in *Campylobacter* population exhibiting a multi-host profile, suggesting the persistence of this foodborne pathogen in different reservoirs and consequently recommended the need to investigate their survival strategy at a higher resolution.

The role of wild boars as reservoir of multi-drug resistant (MDR) *Campylobacter* species in Italy has been recently elucidated by Marotta et al. through application of whole genome multilocus sequence typing (wgMLST) and genomic AMR determinants characterization. Furthermore, comparison of different genomes from guinea pigs (*Cavia porcellus*) by Parker et al. demonstrated novel genomic alterations, including gene gain and loss, that could be associated with guinea pig host specialization related to guinea pig anatomy, dietary intake, and physiology potentially allowing niche adaptation in this animal species. Furthermore, Davies et al. employed a comparative genomics study using WGS analysis complemented with transcriptional and phenotypic variation within epidemiologically related *C. jejuni* isolates from a waterborne outbreak to highlight the role of water-borne infection. This demonstrated a higher pathogenic potential as revealed by the highest levels of virulence gene expression, adhesion to epithelial cells and interleukin 8 (IL-8) induction. The study provided further evidence of bacterial changes due to niche adaptation in the host and/or the environment.

The advent of next generation sequencing (NGS) technologies enabled Song et al. to conduct metagenomics analysis of wild mice (*Micromys minutus*) gut microbiota. The study provided important insights onto the potential role of wild mice as reservoir for *Campylobacter* transmission with higher sensitivity of detection compared to culture-dependent methods due to ability of WGS to determine VBNC *Campylobacter* species.

Finally, despite intensive research, the mechanisms facilitating *Campylobacter* adaptation and factors influencing the survival in the environment are still unclear. An important factor is that the Czech Republic is among the countries with the highest incidence of the disease. Davies et al. focused on waterborne isolates, as there have been more publications recently on waterborne outbreaks of campylobacteriosis. As far as we know, biofilm formation experiments were mostly conducted on isolates originating from various animal, food, and clinical samples, but excluded environmental isolates. However, the phenotypic features of these isolates might be different due to stress conditions in the environment; in turn, they can help bacteria to survive in some way. Then, findings published in this paper illustrate the necessity for future comprehensive studies of waterborne isolates, as they can transmit infection to the same extent, as isolates from meat or clinical isolates.

## Conclusions and Future Directions

*Campylobacter* studies presented in this *Frontiers Research Topic* are linked to the following groups: (1) genome sequencing; (2) metagenomics; (3) different mechanisms of *Campylobacter* survival, and (4) biofilm formation and pathogenesis. Biofilm formation and interaction with other bacteria can also have an influence on bacterial survival outside the host. Therefore, comparing the ecology of *Campylobacter* in different environments provides a better understanding of the infectivity of surviving bacteria to humans. Implementing high-throughput technologies, such as genome sequencing in this context, allows a better understanding of the variations in survival strategies among different *Campylobacter* strains. This combined approach underlines the need to clarify the direct and indirect role of *Campylobacter* ecology in the transmission of *Campylobacter* to humans.

## Author Contributions

All authors contributed to conception and design of the study. All authors wrote sections of the manuscript. All authors contributed to the article and approved the submitted version.

## Funding

Some research resources reviewed in this article were funded by Zewail City internal research grant fund (ZC 004-2019) and joint ASRT/BA research grant (Project number 1110 ) awarded to Dr. Mohamed Elhadidy.

## Conflict of Interest

The authors declare that the research was conducted in the absence of any commercial or financial relationships that could be construed as a potential conflict of interest.

## Publisher’s Note

All claims expressed in this article are solely those of the authors and do not necessarily represent those of their affiliated organizations, or those of the publisher, the editors and the reviewers. Any product that may be evaluated in this article, or claim that may be made by its manufacturer, is not guaranteed or endorsed by the publisher.
